# Landscape of the epigenetic regulation in wound healing

**DOI:** 10.3389/fphys.2022.949498

**Published:** 2022-08-11

**Authors:** Honghao Yu, Yichen Wang, Dawei Wang, Yi Yi, Zeming Liu, Min Wu, Yiping Wu, Qi Zhang

**Affiliations:** Department of Plastic and Cosmetic Surgery, Tongji Hospital, Tongji Medical College, Huazhong University of Science and Technology, Wuhan, China

**Keywords:** wound healing, inflammation, epigenetic regulation, DNA methylation, histone modification, non-coding RNA

## Abstract

Wound healing after skin injury is a dynamic and highly coordinated process involving a well-orchestrated series of phases, including hemostasis, inflammation, proliferation, and tissue remodeling. Epigenetic regulation refers to genome-wide molecular events, including DNA methylation, histone modification, and non-coding RNA regulation, represented by microRNA (miRNA), long noncoding RNA (lncRNA), and circular RNA (circRNA). Epigenetic regulation is pervasively occurred in the genome and emerges as a new role in gene expression at the post-transcriptional level. Currently, it is well-recognized that epigenetic factors are determinants in regulating gene expression patterns, and may provide evolutionary mechanisms that influence the wound microenvironments and the entire healing course. Therefore, this review aims to comprehensively summarize the emerging roles and mechanisms of epigenetic remodeling in wound healing. Moreover, we also pose the challenges and future perspectives related to epigenetic modifications in wound healing, which would bring novel insights to accelerated wound healing.

## 1 Introduction

**FIGURE 1:**
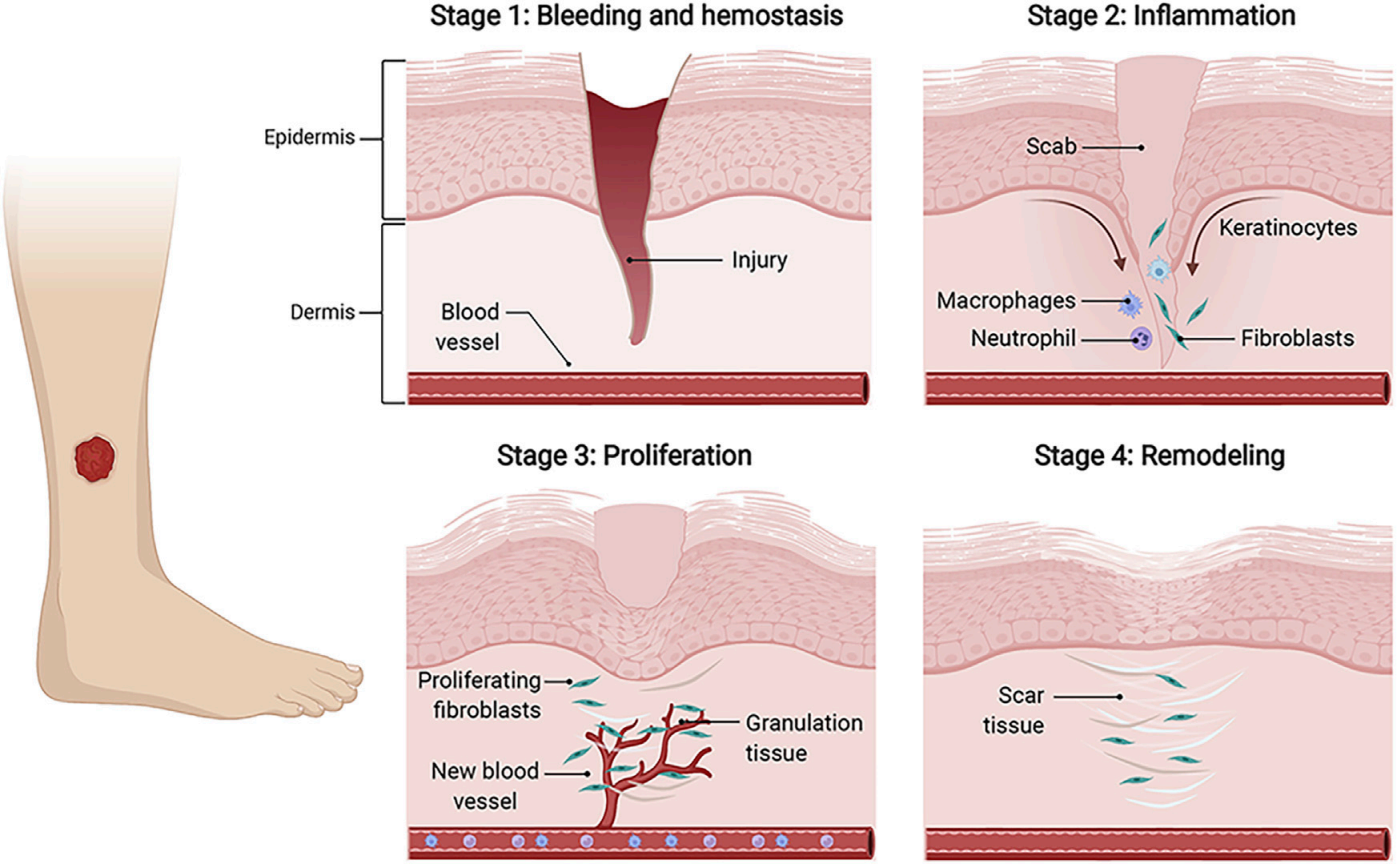
The wound healing process. The four temporally overlapping stages of wound healing mainly include hemostasis, inflammation, proliferation, and tissue remodeling, which involves multiple cells, such as fibroblasts, keratinocytes (KCs), endothelial cells (ECs), and immune cells. Initially, fibrinogens and platelets combine to form the thrombus to stop bleeding, and provide fibrin matrix for infiltrating cells. In the inflammation stage, immune cells, represented by neutrophils and macrophages, are recruited to clear invading pathogens and debris. During the proliferative phase, activated fibroblasts synthesize new ECM and form granulation tissue. KCs migrate to close the wound gap, and ECs migrate to build new blood vessels. Finally, new blood vessels regress, and granulation tissues are remodeled into scar tissues. keratinocytes (KCs), endothelial cells (ECs).

Skin wound healing is an orderly and highly coordinated process aiming to restore skin barrier function (Piperigkou et al., 2018). Generally speaking, the healing process consists of four temporally overlapping phases, including hemostasis, inflammation, proliferation, and tissue remodeling (Gurtner et al., 2008). During these stages, a plethora of distinct cell types dynamically interact with each other and function at specific stages, thus mediating the remodeling healing courses. In the hemostasis phase, injured blood vessels constrict, platelets activate to produce fibrin clots, and immune cells are recruited to the wound areas. In the inflammatory phase, neutrophils and macrophages are involved in the inflammatory response to clear bacteria. In the proliferation stage, vascular endothelial cells participate in the formation of new blood vessels, fibroblasts deposit and remodel the extracellular matrix (ECM), and keratinocytes (KCs) proliferate and migrate to close wounds. During the remodeling phase, cells in the granulation tissue undergo apoptosis, and macrophages break down excessive ECM and apoptotic cells (Rodrigues et al., 2019). (Figure 1).

Minor wounds in healthy people generally heal well, however, large areas, age, infection, diabetes, vascular diseases, and many other factors may delay wound healing, resulting in long-term sequelae. Delayed wound healing brings heavy health and economic burden to patients and society (Eming et al., 2014). Currently, several treatments, such as wound dressings, tissue-engineered substitutes, and skin autografts are available for wound healing (Rousselle et al., 2019). But their effectiveness is mediocre. Therefore, it is urgent to understand the biological mechanisms and develop more effective treatments for wound healing.

Epigenetics refers to heritable changes in gene function without altering the nucleotide sequence. In recent years, epigenetics has been linked to several complex diseases, including cancer, fibrosis, and diabetes (Ling and Rönn, 2019). Epigenetics mainly includes three main mechanisms, DNA modification, histone modification, and regulation of non-coding RNAs (ncRNAs) (Zeybel et al., 2013). These mechanisms interact to regulate gene expression abundance at specific times and places. Precise epigenetic regulation is crucial for maintaining skin homeostasis and shaping the processes of wound healing. Epigenetic dysregulation caused by diabetes and other factors contributes to delayed wound healing and epigenetic modification targeting specific genes could improve the wound healing effect.

The epigenetic mechanism in the wound healing process is still not well understood. Here, this review summarized the presently available studies to investigate the epigenetic modification functioned during wound healing course, mainly including DNA methylation, histone modification, and regulation of ncRNAs represented by microRNA (miRNA), long noncoding RNA (lncRNA), and circular RNA (circRNA). The ncRNAs transfer mediated by extracellular vesicles (EVs) is also an attractive regulation mechanism by affecting post-transcriptional gene regulation to impact the healing process. Also, we discussed the potential epigenetic targets for promoting wound healing. Regulating the wound healing process through precise epigenetic regulation might bring novel insights to accelerated wound healing.

## 2 Mechanisms of DNA methylation in wound healing

DNA methylation refers to the transfer of a methyl group to the C5 position of cytosine in CpG islands under the catalysis of DNA methylation transferase (Moore et al., 2013). DNA methyltransferase-1 (DNMT1), DNMT3A, and DNMT3B are the main methyltransferase enzymes involved in DNA methylation. DNMT3A and DNMT3B mainly play a key role in *de novo* methylation, and DNMT1 is responsible for maintaining the methylation state. DNMT3A and DNMT3B transfer a methyl group to the cytosine position of CpG dinucleotides, and form 5-methylcytosine (5-mC). Besides, the ten-eleven translocation (TET) family is responsible for DNA demethylation. TET converts 5-mC to 5-hydroxymethylcytosine (5-hmC), 5-formylcytosine (5-fC) and 5-carboxycytosine (5-caC) (Niehrs and Calkhoven, 2020). Methylation of CpG islands in promoters can directly block binding of transcription factors to silence transcription, and also acts synergically with other molecules, such as histone modification enzymes, to inhibit transcription (Li and Zhang, 2014). Luo et al. suggested that in the process of corneal epithelial wound healing, the expression of DNMT1 and DNMT3B were up-regulated resulting in increased global DNA methylation levels. This suggested that DNMTs were activated in the wound process (Luo et al., 2019). Besides, Sen et al. showed that DNMT1 was enriched in epidermal progenitor cells, which were required to preserve proliferation endurance and inhibit differentiation. Besides, the differentiated genes, such as LCE3D and S100P, and differentiation-related transcription factors such as POU2F3, MAFF, and SP1, lose methylation during differentiation (Sen et al., 2010). This indicated that DNA methylation promoted the proliferation of epidermal stem cells during tissue repair, and demethylation may indeed lead to premature differentiation.

DNA methylation is implicated in all aspects of wound repair, including cell proliferation, collagen deposition, and angiogenesis. During the transition from scarless to normal healing, the genes involved in the inflammatory response and hyaluronic acid degradation showed increased DNA methylation before the transition, while genes associated with embryonic morphogenesis, synapse functions, and neuron and epithelium development do so after the transition (Podolak-Popinigis et al., 2016). This suggests that DNA methylation mechanisms play an important role in scarless healing.

It reported that diabetic foot fibroblasts (DFFs) and fibroblasts from non-healing diabetic foot ulcers showed lower global DNA methylation and functional annotation identified enrichment of genes associated with angiogenesis and ECM assembly (Park et al., 2014). For example, Singh et al. analyzed the methylation status of toll-like receptor 2 (TLR2) promoter of wounds in 110 human lower extremity wounds, which showed that CpG sites of TLR2 promoter were totally or partially methylated in mostly DFU wound biopsies (Singh et al., 2015). The wound healing impairment caused by diabetes, the dysregulation of hematopoietic stem cells (HSCs) towards macrophage differentiation was dependent on up-regulated DNMT1 expression, accompanied by the hypermethylation of Notch1, PU.1, and Klf4. (Yan et al., 2018). This inflammatory mechanism showed that metabolic disorder induced an epigenetic mechanism in HSCs, which predetermined the gene expression of terminally differentiated inflammatory cells. Gomes et al. found that light-emitting diodes (LED) promoted wound healing, accompanied by increased DNMT3A expression and decreased global DNA methylation in the wound healing process (Gomes et al., 2016). DNA methylation is of great significance for skin wound healing. But DNA methylation occurs on different genes in different cells including macrophages and KCs might have exactly opposite effects on wound healing.

## 3 Mechanisms of histone modification in wound healing

Histone modification usually refers to the modification process of histones, such as methylation, acetylation, phosphorylation, adenylation, ubiquitination, and ADP ribosylation by related enzymes (Audia and Campbell, 2016). Histone modification is a kind of post-translational modification that regulates gene transcription and expression, and histone H3 is the most modified histone. Histone ubiquitination, which is mediated by ubiquitin ligase, is the covalent binding of ubiquitin to lysine residues on histone and non-histone target proteins. Histone ubiquitination plays a key role in regulating transcription and DNA repair (Schwertman et al., 2016). Histone phosphorylation is controlled by two types of enzymes, including kinases, and phosphatases. Histone phosphorylation is mainly involved in DNA damage repair and regulation of transcriptional activity (Healy et al., 2012). Besides, Histone ADP-Ribosylation was mediated by poly (ADP-ribose) polymerase 1 (PARP-1). Recent studies demonstrated that histone ADP-Ribosylation promoted inflammatory gene transcription (Martinez-Zamudio and Ha, 2012). So far, very little studies have confirmed the role of histone ubiquitination, ubiquitination, and ADP ribosylation in wound healing. Histone methylation and acetylation are the most commonly studied mechanisms of wounds (Table 1).

**TABLE 1:** Mechanisms and clinical values of histone modification in wound healing.

Modification classification	Enzymes	Target histone	Mechanism	Clinical value	Ref.
Histone methylation	MLL1	H3K4me3	Up-regulate macrophage-mediated inflammation via NF-kB	Promote wound healing	Kimball et al. (2017)
Histone methylation	MLL1	H3K4me3	Up-regulate TLR4 expression in myeloid cells and control the inflammatory response	Promote wound healing	Davis et al. (2019)
Histone methylation	MLL1	H3K4me3	Up-regulated TLR4 expression in macrophage and increase proinflammatory cytokines expression of macrophage	Impair diabetic wound healing	Davis et al. (2020a)
Histone methylation	Setdb2	H3K9me3	Inhibit the production of NF-κB-mediated inflammatory cytokines and XO-mediated UA in macrophages	Regulate normal transition of macrophage phenotype and improve wound healing	Kimball et al. (2019)
Histone demethylation	N/A	H3K9me2 and H3K27me3	Reverse diabetes-induced histone methylation of the ER β promoter and suppression of NRF1 and SOD2	Epigenetic modification of HSC could be used to promote wound healing.	Xie et al. (2021)
Histone demethylation	Jmjd3	H3K27me3	Shape programmed macrophages toward a proinflammatory phenotype	Impair wound healing	Gallagher et al. (2015)
Histone demethylation	Jmjd3	H3K27me3	Up-regulate NFκB-mediated inflammatory genes in macrophage	Shape macrophage toward proinflammatory state and impair diabetic wound healing	Davis et al. (2020b)
Histone methylation	PcGs	H3K27me3	Silence repair genes including Myc and Egfr	Inhibit wound re-epithelialization	Shaw and Martin, (2009)
Histone demethylation	JMJD3	H3K27me3	Enhance keratinocyte migration by increasing Notch 1 gene expression	Accelerate wound healing.	Na et al. (2017)
Histone methylation	SETD2	H3K36me3	Inhibit keratinocyte proliferation and migration via down-regulate AKT/mTOR signalling	Impair wound healing	Li et al. (2021a)
Histones acetylation	N/A	H3K9ac	Accelerate epithelial migration and active terminal differentiation of KC stem cells	PBMT could accelerate wound healing by up-regulating histones acetylation	Martins et al. (2021)
Histone acetylation	MOF	H4K16ac	Promote NF-κB-mediated inflammatory gene transcription in diabetic wound macrophages	MOF-knockout might accelerate diabetic wound healing	den Dekker et al. (2020)
Histone deacetylation	HDACs	N/A	Increase proinflammatory Ly6Chigh monocytes	HDAC inhibitor could increase repairing Ly6Clow subsets and enhance wound healing	Cabanel et al. (2019)
Histone deacetylation	HDAC6	N/A	HDAC6 inhibitor-TSA hydrogel inhibited IL-1β secretion of macrophages and up-regulated IL-10 level	TSA could accelerate wound healing	Karnam et al. (2020)

### 3.1 Histone methylation

Histone methylation usually occurs at different sites in histones, primarily at lysine and arginine residues, and can be controlled by multiple regulators to activate or inhibit transcription (Jambhekar et al., 2019). Histone methyltransferase (HMT) and demethylase (HDM) are two enzymes important in regulating histone methylation (Yang et al., 2020a). They catalyze methylation of histone tail residues to promote or inhibit transcription. Among them, mixed-lineage leukemia 1 (MLL1), Setdb2, PcGs, SETD2, and Jmjd3 were widely studied in wound healing. Macrophages are crucial participants in maintaining cell homeostasis and regulating the immune response. The epigenetic perturbation of tissue macrophage properties can affect the secretion function and chemotaxis of the macrophages and help modify the process of wound healing. The depletion or overaction of tissue-resident macrophages would result in impaired wound healing. Histone methylation plays an important role in epigenetic regulation driven by macrophages and microenvironmental factors. For instance, the hyperglycemic environment activated Jumonji domain-containing protein 3 (JMJD3) to demethylate trimethylation of lysine 27 on H3 (H3K27me3) and increased interleukin-12 (IL-12) expression in bone marrow progenitor cells in inflammation phase (Gallagher et al., 2015). These epigenetic changes programmed macrophages toward a proinflammatory phenotype in peripheral wounds. These epigenetically “pre-programmed” macrophages result in poised macrophages in peripheral tissue and negatively impact wound repair.

The knockout of MLL1 in mice increased H3K4me3 at nuclear factor-kappa B (NF-κB) resulting in inhibition of inflammatory cytokines production of macrophages in the resolution of the inflammatory phase and also showed promoted wound healing (Kimball et al., 2017). Besides, Davis et al. also suggested that MLL1 increased H3K4me3 on the TLR4 promoter to up-regulate TLR4 expression in macrophages, which resulted in increased pro-inflammatory cytokines expression of macrophages in the inflammation phase and impaired diabetic wound healing (Davis et al., 2019) (Davis et al., 2020a). They further confirmed that palmitate could induce TLR4/MyD88-dependent up-regulation of histone demethylase JMJD3, resulting in the removal of H3K27me3 mark to up-regulate NF-κB-mediated inflammatory genes in macrophage, which shifted macrophage toward pro-inflammatory state and led to impaired diabetic wound healing (Davis et al., 2020b). In the inflammation phase of normal wounds, IFNβ-induced high expression of Setdb2 trimethylated H3K9 inhibited the production of NF-κB-mediated inflammatory cytokines and xanthine oxidase (XO)-mediated uric acid (UA) pathway of purine catabolism in macrophages (Kimball et al., 2019). But diabetes impaired the interferon (IFN) β-Setdb2 axis resulting in a persistent inflammatory macrophage phenotype and then delayed wound healing. Xie et al. showed that the bone marrow transplantation (BMT) of pterostilbene (PTE)-treated diabetic HSCs could reverse diabetes-induced histone methylation of the estrogen receptor (ER) β promoter and suppression of its target genes NRF1 and SOD2 to ameliorate oxidative stress, mitochondria dysfunction, and up-regulated proinflammatory cytokines in peripheral blood mononuclear cells (PBMCs) and macrophages (Xie et al., 2021). Thus epigenetic modification of HSC could be transferred to PBMCs and macrophages by differentiation and then influence wound healing.

It is gradually clear that histone methylation plays an essential role in determining the biological behavior of KC movement and epithelial regeneration. During the wound healing process, PcGs, a polycomb family of proteins, could silence repair genes including Myc and Egfr by laying down H3K27me3, resulting in wound re-epithelialization inhibition (Shaw and Martin, 2009). SETD2 was the histone H3K36 tri-methylase, and SETD2 knockdown promoted KC proliferation and migration *in vitro* and accelerated re-epithelialization and wound healing via activating AKT/mTOR signaling (Li et al., 2021a). Na et al. indicated that during the wound healing course, the up-regulated JMJD3 and NF-κB promoted NOTCH1 gene expression via demethylation of H3K27me3 (Na et al., 2017). Besides, by increasing RhoU and PLAU gene expression, NOTCH1 enhanced KC migration and then accelerated wound healing.

### 3.2 Histone acetylation

Histone acetylation is a key post-translational modification of chromatin structure by opening or closing the chromatin structure. During wound healing, the acetylation of epidermal histone H4 varied over time, including deacetylation of histone H4K5, H4K8, and H4K16 and over acetylation of H4K12 (Nascimento-Filho et al., 2020). It demonstrates that histone modification occurs not only in healing tissue but also in adjacent and distant wound tissue. Histone acetyltransferases (HATs) and histone deacetylases (HDACs) are the two important enzymes that catalyze histone acetylation and deacetylation, respectively. HATs and HDACs have been shown to interact with chromatin remodeling factors and transcription factors to participate in the transcriptional regulation of various physiological and pathological processes (Shen et al., 2015). Abnormal HDAC activity and histone acetylation disruption lead to loss of skin cell function. HDAC activity blockade resulted in elongated F4/80mpositive macrophages *in vitro* and promoted wound healing along with the expansion of repairing Ly6Clow subsets by inducing chromatin remodeling (Cabanel et al., 2019). HDAC6 expression was increased in high-glucose-induced macrophages and diabetic mice wounds (Karnam et al., 2020). Besides, HDAC6 inhibitor tubastatin A (TSA) hydrogel inhibited IL-1β secretion of macrophages and up-regulated IL-10 level to accelerate wound healing. Tumor necrosis factor-α (TNF-α) -dependent HAT males absent on the first (MOF) promoted NF-κB-mediated inflammatory gene transcription in diabetic wound macrophages via H4K16 acetylation in the inflammation phase (den Dekker et al., 2020).

The treatment strategies for targeting histone acetylation may contribute to an accelerated healing phenotype. Histone acetylation and CBP/p300 activation are described as important signaling endpoints involved in cellular plasticity, regulating the expression of genes involved in various biological mechanisms, such as cell proliferation, differentiation, and survival. Martins et al. revealed that wound healing could be accelerated by photobiomodulation therapy (PBMT), which up-regulated the levels of histones acetylation and transcription cofactors CBP/p300 and reduced the level of methyl-CpG-binding domain2 (MBD2) in epithelial cells, consequently resulting in accelerated chromatin relaxation and epithelial migration (Martins et al., 2021). Simultaneously, PBMT also activated the terminal differentiation of KC stem cells by increasing H3K9ac and CBP/p300. Koko et al. demonstrated that the combined HDACi and paclitaxel therapy could enhance the viability and wound healing ability of human adipose-derived stem cells (hADSCs) and simultaneously maintained cytotoxicity toward breast cancer (BC) after breast cancer resections (Koko et al., 2017). Marcotte et al. suggested that the combined suberoylanilide hydroxamic acid (SAHA), a histone deacetylase inhibitor, and paclitaxel therapy reduced the paclitaxel-mediated impairment on wound healing and improved the therapeutic effect of breast cancer (Marcotte et al., 2018).

## 4 Mechanisms of ncRNA in wound healing

### 4.1 MiRNA

MiRNAs are short and highly conserved ncRNA molecules that regulate target gene expression at a post-transcriptional level. The regulation of miRNAs is associated with delayed wound healing by impacting fibroblast behaviors, KC behaviors, inflammation regulation, and angiogenesis.

#### 4.1.1 MiRNA in fibroblast behaviors

Fibroblasts are very heterogeneous cells whose lineage, phenotype, and plasticity are closely related to wound healing outcomes (Mascharak et al., 2020). Dermal fibroblasts are the core participants in the process of wound healing from pro-inflammatory to anti-inflammatory to repair. Dermal fibroblasts are involved not only in the synthesis and remodeling of ECM proteins, but also in the regulation of immune cells, KCs, ECs, and mast cells by secreting a variety of signaling molecules (Stunova and Vistejnova, 2018). Zhou et al. proved that miR-200b decreased proliferation, migration, and collagen formation of fibroblasts via down-regulating Zeb1 expression and then damaged wound healing (Zhou et al., 2019a). Madhyastha et al. indicated that miR-21 expression was increased and significantly decreased during diabetic wound healing (Madhyastha et al., 2012). Meanwhile, miR-21 promoted fibroblast migration *in vitro*, suggesting that miR-21 might be an important regulatory factor in non-healing diabetic wounds. Li et al.reported that knockdown of miR-378a accelerated migration and differentiation of fibroblast and angiogenesis to accelerated wound healing by up-regulating vimentin and integrin β3 (Li et al., 2014). This study showed that miR-378a could interfere with multiple pathways to regulate wound healing. miR-132 enhanced migration of human dermal fibroblast (HDF) by down-regulating RASA1 expression during the wound healing course (Li et al., 2017). Wu et al. showed that D-glucose stimulation suppressed miR-21-3p expression in fibroblasts, while miR-21-3p agonist promoted fibroblast proliferation, collagen synthesis, and growth factor release by inhibiting SPRY1 to accelerate diabetic wound healing (Wu et al., 2020a).

#### 4.1.2 MiRNA in KC behaviors

KCs not only provide structural support, but also interact with other cells, and exert important immunomodulatory effects during wound repair. As the most dominant cell type in the skin, there are versatile roles of miRNAs in KCs in critical stages of wound healing (Soliman et al., 2018). The understanding of the multifaceted roles of KCs in chronic wound pathology might pave a new avenue to accelerate wound healing.

Transforming growth factor-β (TGF-β)-miRNA “see-saw” might be an important regulatory switch in wound healing. Sundaram et al. indicated that miR-198 inhibited KC migration and re-epithelialization during wound healing course via inhibiting DIAPH1, PLAUand LAMC2 (Sundaram et al., 2013). Besides, TGF-β1 could down-regulate KSRP to control a single primary transcript of miR-198 and FSTL1 and then promote FSTL1 expression and suppress miR-198 expression. miR-23b expression could be elevated by TGF-β1 in HaCaT cells and promoted KC migration through down-regulating TIMP3 (Hu et al., 2020a). miR-26a expression decreased after TGF-β1 treatment in HaCaT and human epidermal keratinocytes (NHEK) cells (Jiang et al., 2020a). The miR-26a overexpression restrained KCs migration by decreasing ITGA5 expression and activating the PI3K/AKT pathway, highlighting its suppressive role.

Aunin et al. performed global miRNA profiling of wound skin from young and aged mice, finding that the expression of miR-200c was increased in the aged group (Aunin et al., 2017). Then they found that miR-200c could compromise wound repair in aged skin by inhibiting KC migration and interfering with KC differentiation. Tang et al.suggested that hypoxia in KCs resulted in increased miR-219-5p and decreased TMEM98 (Tang and Ran, 2018). Meanwhile, miR-219-5p could target TMEM98 to inhibit the beneficial effect of TMEM98 in wound healing, including promoting cell proliferation and migration and inhibiting the inflammatory response. miR-126 promoted KC proliferation and migration by down-regulating PLK2 and activating PI3K/AKT signaling pathway (Chang et al., 2019). The overexpression of let-7b inhibited the migration of KCs *in vivo* and delayed wound healing in mice by targeting IGF2BP2 (Wu et al., 2017). miR-96-5p overexpression decreased human primary KC proliferation and migration *in vitro* via suppressing BNIP3/FAK pathway, which indicated that miR-96-5p could modulate KCs to delay wound healing (Wu et al., 2019). miR-93-3p induced proliferation and migration of KCs through inactivating ZFP36L1 and then up-regulating ZFX (Feng et al., 2021). It suggested that miR-93-3p/ZFP36L1/ZFX axis might be a crucial regulator to active KCs in accelerated wound healing. Tymen et al. found down-regulated miR-99 family members in wound skin (Jin et al., 2013). miR-99 family promoted KC proliferation and migration and accelerated wound healing via PI3K/AKT and mTOR pathways. Yang et al. indicated that miR-21 expression in KCs could be up-regulated by TGF-β1, and miR-21 overexpression promoted KC migration and re-epithelialization via decreasing TIMP3 and TIAM1 levels during the wound healing course (Yang et al., 2011). miR-210 was associated with impairment of KC proliferation and migration to damage wound healing (Dallas et al., 2019). Locally injected miR-210 mimics increased granulation tissue, cellular proliferation, and angiogenesis in diabetic wound sets by inhibiting oxygen consumption rate (OCR) and enhancing glycolysis, then decreasing reactive oxygen species (ROS) levels to restore the metabolic balance (Narayanan et al., 2020).

miR-132 expression of KCs was dynamically increased in the inflammatory phase and peaked in the subsequent proliferative phase (Li et al., 2015). Then, they proved that miR-132 promoted KC proliferation via activating the STAT3 and ERK pathways and alleviated inflammation by suppressing the NF-κB pathway. miR-149 overexpression in KCs promoted wound healing by down-regulating IL-1α, IL-1β, and IL-6 and improving the arrangement of collagen fibers, indicating that miR-149 might play an important role in scarless wound healing by reducing inflammation in wound sets (Lang et al., 2017). miR-31 expression of KC was up-regulated by NF-κB and STAT3 pathways in the inflammatory phase and peaked in the proliferative phase (Shi et al., 2018). miR-34a and miR-34c were overexpressed in KCs of venous ulcers (VUs), and miR-34 increased proinflammatory cytokines and chemokines produced by KCs by inhibiting LGR4 in the inflammation phase to damage wound healing (Wu et al., 2020b).

#### 4.1.3 MiRNA in inflammation regulation

As potential biomarkers for inflammatory and metabolic diseases, miRNAs can be used as specific tools to wound prognosis and treat immunological conditions. miRNAs are important players in regulating inflammation-related signaling pathways and reshaping the behavior of immune cells (Boon, 2015). Umehara et al. used a microarray to perform miRNA profiling of neutrophils from bone marrow in diabetic mice and found that miR-129-2-3p was down-regulated in diabetic-derived neutrophils (Umehara et al., 2019). miR-21 overexpressed in the inflammation phase could promote dendritic cell (DC) differentiation *in vitro* and increase the proportion of DCs in rats by inhibiting PTEN to activate AKT/PI3K signaling pathway (Han et al., 2017). It indicated that miR-21 impacted profoundly on DC quantities contributing to accelerated wound healing.

Aggressive inflammation fights off pathogens and removes dead tissue, but excessive or prolonged inflammation can lead to scarring. miRNA has become a key factor in wound healing, and balancing the inflammatory cascade is a challenging task in wound healing. In diabetic wounds, increased TGF-β inhibited DNMT3b-mediated hypermethylation by up-regulating miR-29b to increase COX-2/PGE2 production (Davis et al., 2020c). Overexpression of miR-129-2-3p could inhibit Casp6 and Ccr2 to regulate the function of neutrophils and then promote diabetic wound healing (Umehara et al., 2019). miR-497 expression was reduced in wound skin of diabetic mice, and the intradermal injection of miR-497 accelerated wound healing by down-regulating pro-inflammatory cytokines IL-1β, IL-6, and TNF-α (Ban et al., 2020). Xu et al. reported that miR-146a expression was decreased in diabetic wounds along with up-regulated pro-inflammatory expression, while mesenchymal stem cells (MSCs) treatment decreased wound inflammation and promoted diabetic wound healing by increasing miR-146a expression (Xu et al., 2012). Li et al. indicated that overexpressed miR-23b inhibited pro-inflammatory TNF-α, IL-1β, IL-6, and CCL2, and increased anti-inflammatory IL-10, to decrease the infiltration of inflammatory (Li et al., 2018a).

The inhibition could miR-155 can reduce inflammation in wound beds and increase granulation tissue formation, vascularization, and collagen synthesis to promote wound healing (Ye et al., 2017). After miR-155 was knocked out, the number of M2 macrophages in mice increased, and the degree of wound closure increased (van Solingen et al., 2014). Yang et al. demonstrated that locally antagonizing miR-155 could down-regulated IL-1β and TNF-α and up-regulated IL-10 to reduce the inflammatory response in wound set (Yang et al., 2014) (Yang et al., 2017a). Moura et al. reported that miR-155 increased inflammatory cell wound infiltration and down-regulated proliferation and migration of KC or fibroblast by targeting FGF7, indicating that miR-155 inhibition might accelerate diabetic wound healing (Moura et al., 2019). Besides, miR-155 knockout weakened IL-17/IL-9 response in wound healing to reduce T cell-mediated inflammation through targeting c-Maf in T cells (Wangrong et al., 2019). These suggested that miR-155 was the potential target to attenuate inflammation in wound tissue.

#### 4.1.4 MiRNA in angiogenesis

Hyperglycemia and endothelial dysfunction of diabetes can lead to abnormal angiogenesis and lead to a higher risk of diabetic foot ulcers and death. Decreased angiogenesis and blood flow under the wound bed represent a critical aspect in impairment wound healing. In the context of DFU and other chronic venous stasis and pressure ulcers, various miRNA species engaged in vascular insufficiency, angiogenesis, and angiogenesis-related gene transcription (Ozdemir and Feinberg, 2019). Pizzino et al. presented that antagomir of miR-15b and miR-200b increased the number of new blood vessels in diabetic wound sets by up-regulating VEGF, Ang‐1, and its receptor (Pizzino et al., 2018). miR-23c expression was increased in patients with infected DFU and SDF- 1α expression was significantly decreased in patients with type 2 diabetes mellitus (T2DM) and infected DFU (Amin et al., 2020). This indicated that miR-23c might inhibit angiogenesis in diabetic wounds by specifically regulating SDF-1α. Xu et al. used Gene Expression Omnibus (GEO) database to find that PTSD was differentially expressed in diabetic patients, and miR-152-3p was the potential upstream mechanism of PTEN (Xu et al., 2020a). Besides, miR-152-3p inhibition promoted proliferation and angiogenesis of HUVECs and accelerated wound healing by up-regulating PTED. miR-92a inhibitor MRG-110, up-regulated the expression of miR-92a target gene ITGA5 to increase wound reepithelialization, granulation tissue formation, and angiogenesis in DB/DB mice (Gallant-Behm et al., 2018).

Angiogenesis is a cellular process featured by a series of events involving EC migration, invasion, and assembly into capillaries. The well-balanced angiogenesis requires coordinated changes in EC polarity and rearrangement. The migration and activity of ECs and cytokines to facilitate angiogenesis are critical for stimulating angiogenesis in the proinflammatory wound environment (Veith et al., 2019). The overexpression of miR-148b enhanced EC proliferation, migration, and angiogenesis and reduced cytokine-induced epithelial-mesenchymal transition (EMT) *in vitro* via targeting TGF-β2 and SMAD2 (Miscianinov et al., 2018). Besides, delivery of miR-148b to injured skin led to promoted wound vascularization and accelerated closure. These results demonstrated the pivotal regulatory role of miR-148b in EMT and vascularization during the wound healing course. Icli et al. reported that miR-26a expression in punch skin biopsy wounding of DB/DB mice was 3.5 times higher than that of WT mice (Icli et al., 2016). Besides, the inhibition of miR-26a induced angiogenesis by robustly increasing BMP/SMAD1-ID1 signaling in ECs, and altered M1/M2 macrophage in wound.

The treatment based on miRNA regulation, which supports epithelialization and angiogenesis, will contribute to facilitating the development of blood supply. Lucas et al. developed the injected light-activatable antimiR-92a, which decreased the expression of miR-92a to derepress Itga5 and Sirt1, thus resulting in promoted cell proliferation and angiogenesis in the diabetic wound (Lucas et al., 2017). Wang et al. found that miR-27b expression was decreased in bone marrow-derived angiogenic cells (BMACs) from diabetic mice, and miR-27b mimic promoted BMAC therapy on diabetic skin wound closure and rescued impaired BMAC angiogenic function by repressing TSP-1, Sema6A, and p66shc (Wang et al., 2014). The up-regulated miR-195 expression increased microvessel density and down-regulated NLRX1 in granulation tissue after negative-pressure wound therapy (NPWT) treatment, finally accelerating wound healing (Liu et al., 2018). The expression of miR-18a/19a was up-regulated in wound tissue from patients and HUVECs treated by MDT. Meanwhile, miR-18a/19a could promote angiogenesis by declining TSP-1 (Wang et al., 2020a).

#### 4.1.5 Others

The inhibition of miR-124-3p and miR-139-5p caused by porcine acellular dermal matrix (ADM) could improve the viability of epidermal stem cells (ESCs) and prevent ESCs from differentiating into MFB by up-regulating the JAG1 and Notch1 expression (Chen et al., 2020). The overexpression of miR-203 induced by high glucose weakened the number and proliferation of ESCs by inhibiting Notch and Wnt pathways (Liu et al., 2020). miR-1248 expression was down-regulated in hADSCs of diabetes mellitus (DM) patients, and that hADSCs from DM patients or treated by advanced glycation end products (AGEs) showed reduced treatment efficiency of wound and lower cell proliferation activity (Xiao et al., 2020). miR-21 expression in epithelial cells and mesenchymal cells were up-regulated after a skin injury, and locally antagonizing miR-21 resulted in impaired wound contraction and collagen deposition to delay wound healing (Wang et al., 2012; Li et al., 2018b; Long et al., 2018).

### 4.2 LncRNA

lncRNAs are defined as a type of ncRNA with more than 200 nucleotides in length. By binding to miRNA, lncRNAs can regulate mRNA expression and affect cellular homeostasis, genomic stability, and functional performance (Kopp and Mendell, 2018). The aberrantly expressed lncRNAs have been reported to involve in various disorders, such as cancer, inflammation, and skin regeneration. More than ten lncRNAs have been reported in wound healing.

LncRNA GAS5 is believed to be an anticancer gene in most cancers (Yang et al., 2020b). In wound, lncRNA GAS5 showed a very large stimulative and positive effect. The overexpressed lncRNA GAS5 in diabetic wounds promoted macrophage polarization toward an M1 phenotype through increasing STAT1 (Hu et al., 2020b). Meanwhile, knockdown GAS5 expression promoted the transition of M1-M2 macrophages to rescue impaired wound healing caused by diabetes. Sawaya et al. suggested that topical mevastatin could inhibit c-Myc expression by up-regulating lncRNA GAS5, which accelerated DFU repair by reducing cortisol synthesis in KCs and biopsies from DFU patients (Sawaya et al., 2018). He et al. indicated lncRNA-GAS5 was down-regulated by diabetes and overexpression of lncRNA-GAS5 promoted lymphangiogenesis and accelerated diabetic wound healing via miR-217/Prox1 axis (He et al., 2021a). Notably, lncRNA-ANRIL was decreased in diabetes and could sponge miR-181a to up-regulate Prox1 and increased ANRIL, or inhibited miR-181a suppressed high glucose (HG) -induced apoptosis of lymphatic endothelial cells (LECs) via caspase pathway, which promoted the lymphatic vessel formation and then accelerated diabetic wound healing (He et al., 2019).

Abnormal expression of lncRNA H19 is associated with complications of DM. Guo et al. suggested that lncRNA H19 expression in fibroblasts from the diabetic wound was increased after the modified preservative fluid-preserved autologous blood transfusion (ABT) treatment. Besides, increased H19 recruited EZH2 to enhance HIF-1α histone H3K4me3 methylation and then up-regulated HIF-1α expression, which finally promoted fibroblast activation and accelerated diabetic wound healing (Guo et al., 2018). LncRNA H19 sponged miR-29b to increase FBN1 expression, which could enhance the proliferation and migration of fibroblast (Li et al., 2021b). Thus, lncRNA H19/miR-29b/FBN1 regulatory loop might be a promising treatment for DFU.

LncRNA WAKMAR1 inhibited methylation of the E2F1 promoter by sequestering DNMTs, and then promoted migration of KCs and re-epithelialization of human *ex vivo* wound model (Li et al., 2019a). Herter et al. suggested that WAKMAR2 induced by TGF-β signaling was down-regulated in human chronic wound-edge. Besides, WAKMAR2 inhibited inflammatory chemokine production of KCs and enhanced KC migration to accelerate wound healing (Herter et al., 2019).

LncRNA HOTAIR expression was up-regulated during burn wound healing (Shi et al., 2020a). Overexpression of HOTAIR could enhance ESC proliferation, stemness, and HOTAIR-overexpressing ESCs promoted re-epithelialization and burn wound healing. The overexpressed linc00174 promoted endothelial cell-mediated angiogenesis to accelerate post-burn wound healing via linc00174-EZH2-ZNF24/Runx1-VEGFA regulatory axis (Huang et al., 2020). Jiang et al. suggested that HOTAIR and SCEL were decreased in burn wound tissues along with increased miR-126 (Jiang et al., 2020b). Besides, HOTAIR increased the expression of SCEL by sponging miR-126 to promote HUVEC proliferation, migration, invasion, tube formation, and apoptosis. Thus HOTAIR/miR-126/SCEL axis might modulate burn wound healing by regulating angiogenesis.

In skin tissues, lncRNA TETILA was up-regulated and induced MMP-9 promoter demethylation to impair diabetic wound healing by regulating the activity and localization of demethylation enzymes Ten-eleven translocation 2 (TET2) (Zhou et al., 2019b). The highly expressed lnc-URIDS in diabetic wounds impaired wound healing and delayed collagen production and deposition by targeting Plod1 (Hu et al., 2020c). Liang et al. indicated that MALAT1 enhanced TGFβ2/SMAD2 signaling pathway, regulated proliferation, and migration of fibroblast, and promoted wound healing by targeting miR-141-3p and up-regulating expression of ZNF217 (Liang et al., 2020).

### 4.3 CircRNA

CircRNAs belong to a large category of endogenous ncRNAs that are structurally connected end to end to form single-chain molecules with a covalently closed loop structure (He et al., 2021b). Functionally, circRNAs can regulate the level of transcriptional and post-transcriptional parental genes by acting as miRNA sponges (Kristensen et al., 2019). CircRNAs are widely present in various tissues and cells under physiological and pathological conditions, possessing typical characteristics, including species diversity, evolutionary conservation, biological stability, and disease specificity. Studies have gradually reported the considerable role of circRNAs in wound healing. The circ-Amotl1 promoted fibroblast proliferation and migration and accelerated wound healing by decreasing miR-17-5p expression and enhancing the nuclear translocation of STAT3 (Yang et al., 2017b). circ_PRKDC was down-regulated during the wound healing process, and knockdown of circ_PRKDC activated the KC migration to accelerate wound healing via miR-31/FBN1 axis (Han et al., 2021). Wang et al. suggested that hsa-circ-0084443 was up-regulated in DFU decreased KC migration and enhanced KC proliferation (Wang et al., 2020b). Meanwhile, the global transcriptomic analysis showed that hsa-circ-0084443 might regulate JAK/STAT, EGFR, PI3K, ERK, and HIF-1 signaling pathways. Sheng-ji Hua-yu formula treatment decreased TNF-α, IL-1β, and IL-6 production in diabetic wound tissues. CircRNA-Krt13/miR-665-3p/Itga3 and circRNA-Krt14/miR-706/Mylk4 pathways might play an important role in SJHY formula-mediated diabetic wound healing (Xiang et al., 2021).

## 5 EV-mediated cellular interaction

EVs are naturally occurring vesicles with a double lipid bilayer, secreted by most cell types and of endocytic or membrane-derived origin (Tkach and Théry, 2016). According to their diameter sizes, EVs comprise three main subtypes, exosomes, microvesicles, and apoptotic bodies. The ncRNAs encapsulated in EVs are crucial information interaction mediums as tissue repair mediators and are the most studied epigenetic components in wounds (Table 2).

**TABLE 2:** The epigenetic regulation and mechanisms of EVs in wound healing.

Exosomal contents	Source	Recipient cells	Mechanisms	Biological function	Ref.
miR-27b	hUC-MSCs	HaCaT cells and HSFs	Target ITCH/JUNB/IRE1α axis	Improve proliferation and migration of HaCaT cells and HSFs	Cheng et al. (2020)
miR-21-5p	Mag-BMSCs	HUVECs and HSFs	Inhibit SPRY2 and activate PI3K/AKT and ERK1/2 Pathways	Accelerate wound healing	Wu et al. (2020c)
miR-135a	Human amnion MSCs	Human fibroblast BJ-1 cells	Down-regulate LATS2 levels	Promote BJ-1 cells migration	Gao et al. (2020)
miR-486-5p	ADSCs	HSFs and HMECs	Regulate the Sp5/CCND2	Promote HSF proliferation, migration, and HMEC angiogenesis	Lu et al. (2020)
miR-425-5p miR-142-3p	EPSCs	HDFs	Down-regulate the expression of TGF-β1	Inhibit myofibroblast differentiation	Duan et al. (2020)
miR-126 miR-130a miR-132 miR124a miR-125b miR-21	Human circulating fibrocytes	Diabetic KCs	N/A	Promote migration and proliferation of diabetic keratinocytes and enhance angiogenesis	Geiger et al. (2015)
miR-21-3p	hUC-blood plasma	HSFs and HMECs	Inhibit PTEN and SPRY1	Promote HSF proliferation, migration, and HMEC angiogenesis	Hu et al. (2018)
miR-16-5p	Induced pluripotent stem cells	HaCaT cells	Activate p38/MARK pathway by targeting Desmoglein 3	Promote deep second-degree burn wound healing	Yan et al. (2020)
miR-21	HEKa	HFF-1, endothelial cells	Down-regulate PTEN/RECK, and activate MAPK/ERK signaling	Promote fibroblast migration, differentiation, and contraction, enhance angiogenesis and promote the inflammatory response	Li et al. (2019b)
miR-20b-5p	Plasma of diabetic patients	HUVECs	Inhibit of the Wnt9b/β-catenin signaling pathway	Inhibit angiogenesis	Xiong et al. (2020)
miR-20b-5p	Plasma of diabetic patients	HSFs	Suppress VEGFA expression	Suppress HSF proliferation and promote apoptotic death.	Chen et al. (2021)
miR-221-3p	EPCs	N/A	Possibly target AGE-RAGE, p27, and p57	Promote angiogenesis and cell proliferation	Xu et al. (2020b)
miR-106b	HUVECs	LL29 fibroblast cells and HaCaT keratinocytes	Inhibit JMJD3 and RIPK3	Reduce adhesion and viability of fibroblasts and keratinocytes and inhibit collagen I content and angiogenesis	Qi et al. (2021)
miR-24-3p	Plasma of diabetic patients	HUVECs	Down-regulate PIK3R3 expression	Inhibit angiogenesis, survival, and migration of HUVECs	Xu et al. (2020c)
lncRNA H19	MSCs	Fibroblasts	Sponge miR-152-3p and up-regulated PTEN to inhibit PI3K/AKT signaling pathway	Prevent the apoptosis and inflammation of fibroblasts	Li et al. (2020)
lncRNA-H19	EMNVs	HMEC-1	Impair PI3K/AKT signaling pathway	Promote EC proliferation, migration, and angiogenesis	Tao et al. (2018)
MALAT1	ADSCs	HaCaT and HDF cells	Sponge miR-124 and activate Wnt/β-catenin pathway	Promote the proliferation and migration of HaCaT and HDF cells	He et al. (2020)
has_circ_0075932	Adipocytes	Dermal KCs	Bind with PUM2 and up-regulating AuroraA/NF-kB pathway	Promote inflammation and apoptosis in keratinocytes to impair wound healing	Zhang et al. (2019)
mmu_circ_0000250	ADSCs	EPCs	Induce miR-128-3p/SIRT1-mediated autophagy	Promote EPC proliferation and angiogenesis to accelerate diabetic wound healing	Shi et al. (2020b)

### 5.1 EV-miRNA

By encapsulating multiple bioactive cargoes, EVs derived from stem cells are endowed with huge potency in tissue regeneration by promoting cell migration and proliferation, inducing neovascularization, and regulating the repair environment. The miRNAs in stem cell-derived EVs have positive significance for wound healing. For instance, Cheng et al. indicated that human umbilical cord (hUC) MSC-derived EVs could transfer miR-27b to improve the proliferation and migration of HaCaT cells and human skin fibroblasts (HSFs) *in vitro*, and then accelerate wound healing via ITCH/JUNB/IRE1α axis (Cheng et al., 2020). Wu et al. used Fe3O4 nanoparticles combined with static magnetic field (SMF) to stimulate BMSCs secreting mag-BMSC-Exos, which contained miR-21-5p and accelerated wound healing via inhibiting SPRY2 and activating PI3K/AKT and ERK1/2 pathways (Wu et al., 2020c). The miR-135a produced by ADSC-Exos enhanced fibroblast migration and then accelerated wound healing in rats by down-regulating LATS2 levels (Gao et al., 2020). In addition, ADSC-EVs containing miR-486-5p promoted HSF proliferation, migration, and human microvascular endothelial cells (HMECs) angiogenesis, resulting in accelerated wound healing via regulating the Sp5/CCND2 (Lu et al., 2020). Epidermal stem cells (EPSC)-Exos could accelerate wound healing and prevent scar formation (Duan et al., 2020). In the process, miR-425-5p and miR-142-3p from EPSC-Exos down-regulated TGF-β1 expression to inhibit myofibroblast differentiation and collagen I deposition. Moreover, induced pluripotent stem cells (iPSCs)-MVs contained up-regulated miR-16-5p, which promoted KCs migration by activating the p38/MARK pathway by targeting Dsg3, leading to accelerated mice deep second-degree burn wound healing (Yan et al., 2020). These results suggest that the EV-miRNAs derived from hUC-MSCs, BMSCs, hADSC, EPSC, and iPSCs, exhibit excellent capability in stem cell-based therapeutic options.

EV-miRNAs are involved in the cellular interaction, and thus regulate multiple skin cell functions for better or worse healing status. Fibrocyte-derived exosomes promoted the proliferation of diabetic KCs and angiogenesis *in vitro* (Geiger et al., 2015). Furthermore, the *in vivo* results uncovered that fibrocyte-derived exosomes were enriched with miR-21, miR-124a, miR-125b, miR-126, miR-130a, and miR-132, attributing to accelerated diabetic wound healing. Exosomes from human umbilical cord blood plasma (UCB-Exos) up-regulated the proliferation and migration of fibroblasts and the angiogenic activities of ECs *in vitro*, and also promoted re-epithelialization, inhibited scar formation, and enhanced angiogenesis *in vivo* (Hu et al., 2018). Meanwhile, the enriched miR-21-3p in UCB-Exos played a key role in UCB-Exos-mediated wound healing ability via inhibiting PTEN and SPRY1. MV miR-21 from KCs promoted fibroblast functions and skin wound healing via down-regulating PTEN and RECK and activating MAPK/ERK signaling (Li et al., 2019b).

MiR-20b-5p was validated as an enriched miRNA in circulating exosomes isolated from T2DM patients. Mechanismly, miR-20b-5p impaired fibroblast collagen synthesis, proliferation, and angiogenesis by inhibiting the VEGFA expression and Wnt9b/β-catenin signaling pathway, leading to the slowed healing process (Chen et al., 2021) (131). Xu et al. suggested that the highly expressed miR-221-3p in endothelial progenitor cell (EPC) -derived exosomes, possibly targeted AGE-RAGE, p27, and p57 to promote angiogenesis and cell proliferation to accelerate wound healing in diabetic mice (Xu et al., 2020b). EVs from HUVEC with miR-106b overexpression reduced adhesion and viability of fibroblasts and KCs, collagen I content, and angiogenesis to impair wound healing by down-regulating JMJD3 and RIPK3 (Qi et al., 2021). Besides, miR-24-3p was enriched in circulating exosomes derived from diabetes patients, and inhibition of miR-24-3p promoted angiogenesis, survival, and migration of HUVECs via increasing PIK3R3 expression (Xu et al., 2020c).

### 5.2 EV-lncRNA

EV-transmitted lncRNAs function as a communication medium, playing an important role in EV-mediated wound recovery. MSC-released exosomal lncRNA H19 promoted fibroblast proliferation and migration to accelerate the wound healing process of DFU via lncRNA H19/miR-152-3p/PTEN axis (Li et al., 2020). EV-mimetic nanovesicles (EMNVs) with higher content of lncRNA-H19 promoted EC proliferation, migration, and tube formation *in vitro*, and enhanced angiogenesis, re-epithelization, and wound healing *in vivo* via restoring the AKT vitality (Tao et al., 2018). By targeting miR-124 and activating the Wnt/β-catenin pathway, ADSC-Exos containing MALAT1 promoted the proliferation and migration of HaCaT and HDF cells in the skin lesion model impaired by H2O2 (He et al., 2020).

### 5.3 EV-circRNA

EV-circRNA has been a hot area of research currently due to the high abundance, genetic manipulation, stability, and transfer characteristics to recipient cells. Has_circ_0075932 was overexpressed in human normal adipose tissue and burned skin of obese persons. Besides, circ_0075932 from adipose-derived exosomes enhanced inflammation and apoptosis of epidermal KCs by directly binding with PUM2 and up-regulating AuroraA/NF-kB pathway (Zhang et al., 2019). The exosome divided from mmu_circ_0000250-modulated ADSCs decreased HG-induced EPC apoptosis and promoted EPC proliferation and angiogenesis *in vitro*, along with accelerating streptozotocin (STZ)-induced diabetic wound healing *in vivo* by inducing miR-128-3p/SIRT1-mediated autophagy (Shi et al., 2020b).

## 6 Limitations and future perspectives

Here, we have successfully emphasized the emerging epigenetic remodeling in wound healing. Nevertheless, there are still some issues to resolve in this field. Firstly, in terms of mechanism exploration, the current studies concentrate mainly on miRNA-related epigenetic regulation, while lncRNA and circRNA are not thoroughly studied. Determining the role of epigenetics in effect mechanism or therapeutic response remains a challenge in improving wound healing. Single-cell sequencing and spatial multi-omics can be used to evaluate the changes of epigenetics in different cells and spaces during different wound healing stages to achieve precise epigenetic regulation of wound healing. Besides, normal wound healing is a complex multi-stage process involving synergies between fibroblasts, KCs, immune cells, and various factors (Takeo et al., 2015). The intercellular communication tools represented by EVs are a microcosm of this complex network. This makes it a very complex proposition to determine which cells or pathways dominate or even become therapeutic targets during a particular healing phase.

Secondly, at present, the wound models are mainly established in rodent animals, represented by mice and rats. There are significant structural and morphological differences between rodent skin and human skin, which results in the differential wound course and outcome. Hair follicles are more abundant in rats and mice than in humans, and hair follicle stem cells can promote wound healing in rodents (Grada et al., 2018). It means that the testing treatments in animal models cannot fully reproduce the complexity of chronic wounds in humans. Establishing wound models that are more in line with clinical standards, such as organoid models derived from human skin cells, and wound models represented by pig skin, also provides support for real-world research on wound studies.

Thirdly, wound healing is a complex and orderly biological process. As mentioned above, epigenetics can alter the outcome of wound healing by regulating the biological behaviors of key cells in wound healing. Essentially, this means that epigenetic-related regulatory methods, such as small molecule inhibitors, antibodies, or genetic modifications, can effectively regulate cell behavior and wound healing efficiency. Current studies typically use the same epigenetic therapy at different stages of wound healing. According to the characteristics of different wound stages, different epigenetic regulation strategies can be adopted to ensure orderly wound healing. Non-healing wound is often associated with systemic diseases, such as diabetes, and immune deficiency syndrome. Therefore, for wound therapy, the current studies mainly focus on the effect of local epigenetic regulation, and the algorithm combining local and systematic epigenetic regulation may achieve better results. Of course, it is also important to combine epigenetic therapy with a variety of effective routine therapies in treating wounds. Epigenetics not only has a positive effect on wounds, but also may produce synergistic amplification effects when combined with traditional or existing treatment methods such as physical therapy, negative pressure suction, and drug treatment. Lastly, stem cell therapy is proving to be an attractive strategy due to the ability to differentiate in multiple lineages, multiple donor tissue types, and paracrine-related immune regulation (Kosaric et al., 2019). Stem cell exosome cargo has also been shown to be an efficient transporter of protein and genetic material. Using specific epigenetic approaches, it is possible to modulate the secretory expression profile of stem cell exosomes and to intervene in the wound process through various cellular function modulations. Because epigenetic modification can modify the biological behavior and function of stem cells and indirectly regulate wound healing, epigenetic modification of stem cells is also expected to be used in wound healing. More importantly, the existing experiments of epigenetic regulation in treating wound healing, are principally explored and investigated at the cellular level and animals, suggesting that these studies are just undergoing preclinical. This is because the majority of epigenetic modifiers are preliminarily validated in a relatively small number and independent studies. Besides, there is strict supervision, therapeutic, effectiveness, and biosafety requirements in clinical studies, which commits to the long-term road to ultimate success in clinical practice. Future large-sample clinical trials are needed to further clarify the effectiveness of epigenetic regulation in wound healing.

## 7 Conclusion

**FIGURE 2:**
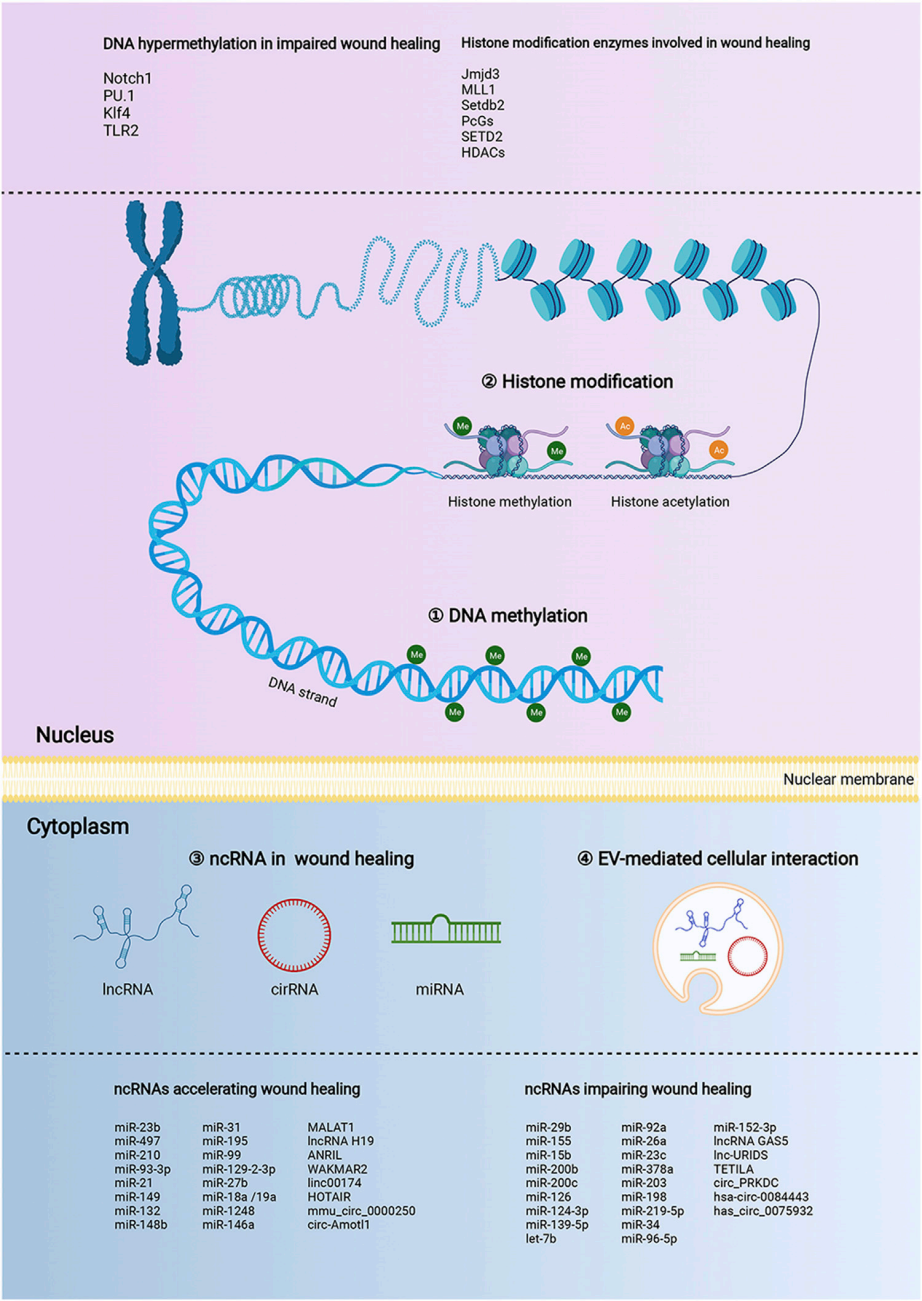
The landscape of the epigenetic regulation in wound healing. The epigenetic modifications of the wound healing process are presented by DNA methylation, histone modifications, and non-coding RNAs (ncRNAs). In the nucleus, the nucleosome is the functional unit of the chromatin, which is made up of DNA and histone proteins. Histone modifications, including methylation (me), acetylation (ac) can regulate the transcription process by affecting the looseness of chromatin. DNA methylation refers to the transfer of a methyl group to the C5 position of cytosine in CpG islands. NcRNAs provide additional epigenetic regulation in the cytoplasm, by regulating mRNA expression at the levels of transcription, RNA processing, and translation. MicroRNA (miRNA), long noncoding RNA (lncRNA), and circular RNA (circRNA) are the major ncRNA types involved in wound healing. NcRNAs enriched and stabilized in EVs mediate skin-related cell communication. The above three regulatory mechanisms could manipulate various functions and fates of multiple would-associated cells, including proliferation, migration, apoptosis, and differentiation, consequently impacting the process of wound healing.

**FIGURE 3:**
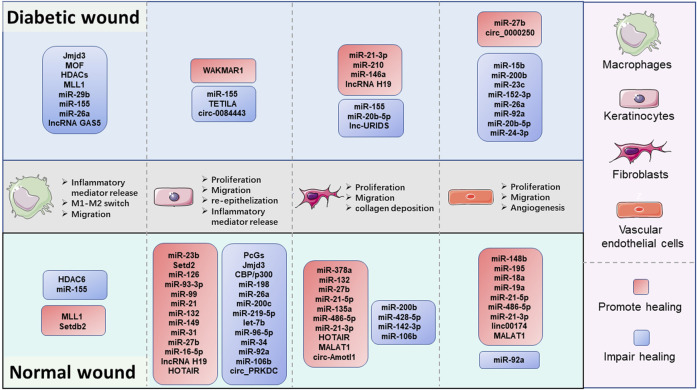
The comparison of epigenetic regulation in pivotal cellular components during the diabetic and normal wound healing process. Macrophages, keratinocytes, fibroblasts, and vascular endothelial cells are the major cellular components in wound repair. Fundamentally, DNA methylation and histone modification mainly regulate the cell function of macrophages, keratinocytes, and non-coding RNA mediate the function regulation of all four types of cells. Epigenetic changes can regulate the secretion of inflammatory factors, M1-M2 switch, and migration in macrophages. Besides, the epigenetic modifications also affect the proliferation migration abilities of keratinocytes, fibroblasts, and vascular endothelial cells to promote or impair the re-epithelialization, collagen deposition, and angiogenesis processes in wound healing.

Collectively, the preponderance of evidence continues to substantiate that epigenetic modifications are key coordinators in wound healing (Figure 2). Especially, the DNA hypermethylation of Notch1, PU.1, Klf4, and TLR2 are crucial orchestrators in impairing the refractory wound process. The abnormal activity or disruption of the histone modification enzyme, including Jmjd3, MLL1, Setdb2, PcGs, SETD2, and HDACs, can influence the growth and function of macrophages and KCs, thus mediating the process of multiple stages of wound healing. ncRNAs, especially miRNAs, are the largest and most studied epigenetic substances that activate or inhibit the expression of target genes. EVs are important tissue repair mediators that transmit epigenetic information in regulating proximal and distal reciprocal action. Some specially ncRNAs (miR-23b, miR-497, miR-210, miR-93-3p, miR-21, miR-149, miR-132, miR-148b, lncRNA H19, MALAT1, and circ-Amotl1), play a role in accelerating wound healing, the other ncRNA types (miR-29b, miR-155, miR-15b, miR-200b, miR-200c, miR-126, miR-124-3p, miR-139-5p, let-7b, lncRNA GAS5, and circ_PRKDC) exert the opposite function by impairing wound healing. The current studies mainly focus on diabetic wounds and normal wounds (Figure 3). Important cellular components in tissue repair represented by macrophages, KCs, fibroblasts, and vascular endothelial cells, are intensively involved in the repair process of diabetic and normal wounds. Specifically, macrophages can be defined as pro-inflammatory macrophages (M1 macrophages) and anti-inflammatory macrophages (M2 macrophages) phenotypes. They are featured by specific inflammatory factor expression profiles, thus participating in the whole process of repair. The epigenetic mechanisms occurred in macrophages, represented by Jmjd3, HDAC6, and MLL1, affect the wound process by regulating inflammatory mediator release, M1-M2 switch, and migration of macrophages. KCs are the dominant cells for repairing damaged skin, and fibroblasts are stromal cells that promote deposition and ECM reconstruction. The proliferation, migration, re-epithelialization, and secretion of inflammatory factors in KCs are regulated by WAKMAR1, miR155, TETILA, etc (diabetic wound) and miR-23b, Setd2, PcGs, etc (normal wound). The fibroblast behaviors, proliferation, migration, and collagen deposition, are regulated by miR-21-3p, miR-155, and lnc-URIDS. Finally, vascular endothelial cells that promote angiogenesis are affected by miR-27b, miR-15b, miR-148b, miR-92a, etc. These cellular behavior alterations can affect wound outcomes (Figure 3). Finally, these molecules involved in epigenetic regulation are compelling candidates as potential diagnostic and prognostic markers for wound healing disorders, as well as promising therapeutic targets for refractory non-healing wounds.

## Glossary

ABT: autologous blood transfusion
AGE: advanced glycation end products
ADP-ribose: poly
BC: breast cancer
BMACs: bone marrow-derived angiogenic cells
BMT: bone marrow transplantation
circRNA: circular RNA
DC: dendritic cell
DNMT1: DNA methyltransferase-1
DFFs: diabetic foot fibroblasts
DM: diabetes mellitus
ESCs: epidermal stem cells
EPSC: Epidermal stem cells
ECM: extracellular matrix
EPSC: Epidermal stem cells: Epidermal stem cells
ER: estrogen receptor
EPC: endothelial progenitor cell
EMNVs: EV-mimetic nanovesicles
EVs: extracellular vesicles
EMT: epithelial-mesenchymal transition
GEO: Gene Expression Omnibus
HATs: Histone acetyltransferases
HDACs: histone deacetylases
H3K27me3: demethylate trimethylation of lysine 27 on H3
HDM: demethylase
HMT: histone methyltransferase
hADSCs: human adipose-derived stem cells
HDF: human dermal fibroblast
HG: high glucose
hUC: human umbilical cord
HSFs: human skin fibroblasts
HMECs: human microvascular endothelial cells
HSCs: hematopoietic stem cells
IL-12: interleukin-12
IFN: interferon
iPSCs: induced pluripotent stem cells
lncRNA: long noncoding RNA
JMJD3: Jumonji domain-containing protein 3
LECs: lymphatic endothelial cells
LED: light-emitting diodes
miRNA: microRNA
MLL1: mixed-lineage leukemia 1
MOF: males absent on the first
MBD2: methyl-CpG-binding domain2
NF-κB: nuclear factor-kappa B
ncRNAs: non-coding RNAs
NHEK: human epidermal keratinocytes
MSCs: mesenchymal stem cells
NPWT: negative-pressure wound therapy
OCR: oxygen consumption rate
PARP-1: polymerase 1
PTE: pterostilbene
PBMCs: peripheral blood mononuclear cells
PBMT: photobiomodulation therapy
ROS: reactive oxygen species
STZ: streptozotocin
SMF: static magnetic field
SAHA: suberoylanilide hydroxamic acid
TET2: Ten-eleven translocation 2
T2DM: type 2 diabetes mellitus
TGF-β: Transforming growth factor-β
TSA: tubastatin A
TNF-α: Tumor necrosis factor-α
TLR2: toll-like receptor 2
KCs: keratinocytes
5-mC: 5-methylcytosine
TET: en-eleven translocation
5-hmC: 5-hydroxymethylcytosine
5-fC: 5-formylcytosine
5-caC: 5-carboxycytosine
UA: uric acid
VUs: venous ulcers
UCB-Exos: Exosomes from human umbilical cord blood plasma
XO: xanthine oxidase.
